# Compassionate Release and COVID-19: Analyzing Inconsistent Applications of the First Step Act by Federal Courts

**DOI:** 10.1017/jme.2025.10127

**Published:** 2025

**Authors:** Helen Mooney, Kayla Larkin, Mara Howard-Williams

**Affiliations:** 1 Yale School of Public Health, Yale University, New Haven, CT; 2 United States Centers for Disease Control and Prevention, Atlanta, GA

**Keywords:** compassionate release, COVID-19, law, incarceration, policy

## Abstract

The COVID-19 pandemic has posed a significant health threat to people in corrections facilities due to communal living, inability to social distance, and high rates of comorbidity among incarcerated populations. Combined with the First Step Act of 2018, which granted incarcerated individuals seeking compassionate release access to the courts, the pandemic increased the number of people in federal prisons petitioning for early release due to health risk. Analysis of federal compassionate release case law throughout the pandemic reveals inconsistent judicial reasoning related to COVID-19-based requests. Inconsistently interpreted compassionate release factors include vaccination status, COVID-19 reinfection, and the “degree” of extraordinary circumstances considered. Varied application among federal districts produced inequitable access to compassionate release. Therefore, this analysis provides insight into how an unclear policy can create disparate public health outcomes and considerations for compassionate release determinations in future times of uncertainty, such as a pandemic.

## Introduction

In accordance with the Sentencing Reform Act of 1984, the United States Federal Bureau of Prisons (BOP) or a federally incarcerated individual can request “compassionate release” if there are “extraordinary and compelling reasons” for the individual’s early release, such as terminal illness.^1^ Because of congregate living in incarcerated settings, the COVID-19 pandemic posed a disproportionate threat to incarcerated people, resulting in an increase in petitions for compassionate release to prevent viral transmission.^2^

In April 2020, as the COVID-19 pandemic swept through congregate living facilities across the United States, Alberto Pena filed for emergency compassionate release from federal prison under 18 U.S.C. § 3582. Pena cited his heightened risk of COVID-19 resulting from hyperlipidemia (high cholesterol) and hypertension as cause for requesting release. In 2016, he pled guilty to a robbery charge and was sentenced to an 84 month sentence at FCI Fort Dix, New Jersey. Under 18 U.S.C. § 3582, incarcerated people must demonstrate “extraordinary and compelling reasons” to justify early release, while also presenting low danger to the public and exhausting administrative requirements. In May 2020, the United States District Court for the Southern District of New York granted Pena’s request, concluding that “Mr. Pena continues to face extraordinary danger from COVID-19 so long as he remains in custody. Mr. Pena suffers from multiple medical conditions that make him especially vulnerable to the virus.”^3^

Just one week after the Pena decision, on May 15, 2020, Chadwick Thompson filed a motion for compassionate release from FCI Texarkana in the United States District Court for the Eastern District of Texas, pursuant to 18 U.S.C. § 3582. Thompson cited his chronic illness and resulting elevated risk of COVID-19 as the reasons warranting release — hypertension, high cholesterol, and a stroke ten years prior. The court denied his petition. Thompson appealed this denial to the United States Court of Appeals for the Fifth Circuit, which again denied his request. Referencing Thompson’s high cholesterol and hypertension, the court reasoned that neither of these conditions rendered Thompson’s circumstances “extraordinary” for the purposes of the statute.^4^

Pena and Thompson filed for release from federal prison citing the same concerns of contracting COVID-19 and the same chronic conditions for elevating their COVID risk. How could these health conditions pose “extraordinary and compelling” reasons for release for Pena, but not for Thompson? Analysis of publicly available federal compassionate release case law throughout the pandemic reveals inconsistencies in judicial reasoning related to COVID-19-based release requests, including vaccination status, COVID-19 reinfection, and the “degree” of extraordinary circumstances. We aim to outline these inconsistencies and their potential public health implications.

## Background

### The History of Compassionate Release and the First Step Act

Compassionate release was first codified in 1984 under the Sentencing Reform Act.^5^ Though compassionate release purported to lower the federal prison population, the BOP rarely approved petitions in the years following the Act. An estimated 24 federally incarcerated individuals received compassionate release between 2006 and 2011,^6^ followed by 6% of the approximately 5,400 petitioners seeking release between 2013 and 2017.^7^ These trends have been thought to result from the arduous petition process and discretion granted to the BOP (which lacked judicial oversight).^8^ Until 2018, people seeking compassionate release required approval from the BOP. Petitioners faced a lengthy four-step approval process requiring the prison warden, Regional Director, BOP Central Office, BOP Medical Director, and Assistant to the US Attorney to recommend their release.^9^

In 2006, the US Sentencing Commission created a “Guidelines Manual” to outline circumstances that qualify for compassionate release. §1B1.13 opened with the qualifier that “upon motion of the Director of the Bureau of Prisons under 18 U.S.C. § 3582(c)(1)(A), the court may reduce a term of imprisonment” if “extraordinary and compelling reasons warrant the reduction” or “the defendant (i) is at least 70 years old; and (ii) has served at least 30 years in prison pursuant to a sentence imposed under 18 U.S.C. § 3559(c) for the offense or offenses for which the defendant is imprisoned.”^10^

The Guidelines Manual has witnessed numerous amendments over the years. Between 2006 and 2021, the title of §1B1.13 excluded the qualifier “Reduction in terms of Imprisonment *as a result of a Motion by Director of Bureau of Prisons*,” and the “Applications Notes” of the statute expanded to characterize extraordinary and compelling reasons for release, including medical conditions (terminal illness, serious conditions, serious impairment, or deterioration), age, family circumstances, and “Other Reasons—as determined by the Director of the Bureau of Prisons.”^11^ Though the Commission expanded compassionate release guidance after 2006, it is important to note that at the pandemic’s inception, §1B1.13 continued to state that compassionate release may be considered “upon motion of the Director of the Bureau of Prisons” and that “Other Reasons” justifying early release may be determined *by the BOP.* ^12^

This language — delegating responsibility for the determination of “other reasons” to the BOP — was important in the context of the First Step Act of 2018, which expanded compassionate release to allow defendants to petition courts directly for release. Per the Act, thirty days after requesting compassionate release from the prison warden, incarcerated people can directly petition federal courts for release under 18 U.S.C. § 3582(c)(1)(A) instead of waiting for BOP approval.^13^ The admission that additional extraordinary and compelling circumstances could qualify a person for release “as determined by the Director of the Bureau of Prisons” produced a judicial divide in the interpretation of whether §1B1.13 allowed courts to determine “other reasons” suitable for release in the wake of the First Step Act.^14^ *United States v. Cantu* and *United States v. Brooker* marked early decisions of this reasoning, discrediting BOP authority by citing the gaps in use and execution of compassionate release in the past. Though most district courts ruled in favor of judicial discretion regarding reasons for release, a minority limited reasons to those explicitly outlined in the Sentencing Commission’s Guidelines manual. *United States v. Lynn* and *United States v. Bryant* marked early decisions of this opinion, though several courts followed suit.^15^

In November 2023, after the pandemic compassionate release cases discussed herein were decided, the Sentencing Commission updated the Guidelines to remove language attributing determination of “other reasons” warranting release to the BOP.^16^ In addition, in the wake of the pandemic, the Commission added the following as a qualifying medical condition for compassionate release:

(D) The defendant presents the following circumstances— (i) the defendant is housed at a correctional facility affected or at imminent risk of being affected by (I) an ongoing outbreak of infectious disease, or (II) an ongoing public health emergency declared by the appropriate federal, state, or local authority; (ii) due to personal health risk factors and custodial status, the defendant is at increased risk of suffering severe medical complications or death as a result of exposure to the ongoing outbreak of infectious disease or the ongoing public health emergency described in clause (i); and (iii) such risk cannot be adequately mitigated in a timely manner.^17^

This language did not apply to cases prior to November 2023, and it was added in the wake of pandemic petitions. However, even this explicit reference to heightened pandemic risk as a factor suitable for compassionate release does not resolve the pervasive inconsistencies in case decisions observed throughout the COVID-19 pandemic.

### Calls for Reform

Legal scholars have outlined numerous suggestions to reform compassionate release policies. Researchers have demonstrated barriers to access the policy, outlining individual-level gaps in knowledge, social support, distrust of medical personnel, overly optimistic medical prognoses, and inadequate planning for release. At the policy level, barriers include the stringent eligibility requirements, difficult application process, and political concern over repeat offenses.^18^ Arguments for reform include calls for greater access to compassionate release for people convicted of violent crimes, people serving long sentences for drug-related crimes, and elderly incarcerated people.^19^ Regarding the First Step Act Guidelines Manual discrepancy, researchers have argued that courts should have the authority to make discretionary compassionate release case determinations.^20^ These proposals include calls for the Sentencing Commission to codify judicial discretion, Congressional reform, or en banc review of the 11th Circuit *United States v. Bryant* case.^21^ Suggestions to expand access to compassionate release include removing public safety reviews from medical eligibility screening, implementing standard definitions related to public safety evaluations, and expanding parameters and educational access for people seeking release.^22^ Scholars increasingly called for compassionate release reform throughout the pandemic. In the context of COVID-19, calls for policy reform propose faster review of petitions and expanded judicial discretion.^23^

### COVID-19: An Unforeseen and Complicating Factor for Federal Compassionate Release

The pandemic posed a disproportionate threat to incarcerated people, far above that of the general population. Due to the high burden of mental and physical illness associated with incarcerated living conditions, over 32% of incarcerated people identify as disabled, increasing their COVID-19 risk.^24^ The United States Centers for Disease Control and Prevention (CDC) has outlined several health conditions that increase a person’s COVID-19 risk, including cancer, diabetes, cardiovascular disease, mental health conditions, HIV, and others.^25^ In addition, communal living conditions found in nearly all incarcerated settings further amplify the spread of communicable diseases and elevate health risk for elderly and immunocompromised people. COVID-19 transmission risk in incarcerated settings depended upon structural factors including ventilation, ability to social distance, and staff changes.^26^ Finally, people of older age and racial and ethnic minorities have faced higher risk of severe COVID-19.^27^

Given these elevated health risks of incarceration, compassionate release petitions increased significantly throughout the COVID-19 pandemic. According to a report by the Marshall Project, approximately 31,000 people in federal prisons petitioned for compassionate release between March 2020 and June 2021. However, only 10% of these petitions yielded release.^28^

## COVID-19 Compassionate Release Cases

At the start of the pandemic, the First Step Act had only recently reformed federal compassionate release policies, which even then faced unanswered judicial questions (as demonstrated by the Guidelines Manual discrepancy).^29^ The pandemic produced new challenges for the policy, as courts had to weigh petitioners’ concern of contracting a communicable illness in the already questionable federal guidance.^30^ The inconsistent application of federal compassionate release law in district and circuit courts throughout the COVID-19 pandemic provides a unique lens to examine the ethical implications of evolving federal compassionate release principles. The Attorney General directed that BOP “prioritize the use of [its] various statutory authorities to grant home confinement for inmates seeking transfer in connection with the ongoing COVID-19 pandemic.”^31^ However, courts stressed that “the rampant spread of the coronavirus and the conditions of confinement in jail, alone, [were] not sufficient grounds to justify a finding of extraordinary and compelling circumstances.”^32^ A US Sentencing Commission review of court opinions indicates that courts considered a wide array of circumstances and factors — including the spread of COVID-19 in the facility, preexisting conditions in the context of CDC COVID-19 risk data, exhaustion of administrative remedies, danger to the public, and length of sentence served when evaluating petitions.^33^ These considerations were outlined in case law reviewed for this study.

We reviewed federal compassionate release case decisions related to COVID-19 risk in the early years of the pandemic. We searched Google Scholar’s database of case law for publicly available federal compassionate release case decisions in which petitioners cited COVID-19 risk in their request for release. We reviewed these materials for the health risks cited by petitioners and court interpretations of pandemic risk factors. The inconsistent application of public health concepts across these petitions raises ethical concerns about compassionate release in the context of the pandemic. Notably, these inconsistencies relate to vaccination status, COVID-19 reinfection, and the “degree” of extraordinary circumstances.

### COVID-19 Vaccines

Widespread vaccination is a key public health strategy to minimize the impact of COVID-19. Vaccination has been well-demonstrated to reduce the incidence and severity of COVID-19 cases,^34^ calling into question court interpretations of vaccination status when determining whether extraordinary and compelling reasons justify compassionate release. Further complicating the issue, many courts have attributed vaccination status as a reason to decline requests for release.

The court in *United States v. Lemons* reasoned that “a defendant’s incarceration during the COVID-19 pandemic—when the defendant has access to the COVID-19 vaccine—does not present an ‘extraordinary and compelling reason’ warranting a sentence reduction.”^35^ The *Lemons* case highlights a significant pandemic controversy: COVID-19 vaccine access for incarcerated people.^36^ The Federal Bureau of Prisons received their first COVID-19 vaccines in December 2020. Facility staff were allowed first access to vaccines, followed by incarcerated people with “high priority” positions in facilities, people with high COVID-19 risk as classified by CDC, people who “may have” high COVID-19 risk as classified by CDC, and other incarcerated people.^37^ Within the Federal Bureau of Prisons, the BOP had offered the vaccine to 100% of prison staff and 70% of prison residents by April 2021. Fifty percent of employees and 64% of incarcerated people had accepted vaccinations at that time.^38^

Some courts have held that the availability of vaccines changes the landscape of COVID-19 compassionate release requests. In *United States v. Reed*, for example, the court determined that “now that COVID-19 vaccinations are being administered throughout the Bureau of Prisons, compassionate release motions generally lack merit.”^39^ These courts have characterized vaccine acceptance as a factor that significantly mitigates the risk of harm, and therefore after vaccination, COVID-19 risk no longer presents extraordinary and compelling circumstances warranting release. In the *United States v. Rodrigues*,^40^ *United States v. Ueki*,^41^ *United States v. Russo*,^42^ *United States v. Burrough*,^43^ *United States v. Sanchez*,^44^ and *United States v. Meza-Acosta* opinions,^45^ courts specifically articulated that vaccination detracts from petitioners’ arguments for extraordinary and compelling circumstances warranting release. In fact, the *United States v. Singh* opinion cites CDC guidelines allowing vaccinated people to gather unmasked indoors as indication that Singh’s vaccination invalidates his argument for release.^46^

By contrast, when petitioners were unvaccinated voluntarily, many courts did not grant release. In *United States v. Reynoso*, the court found that “the sincerity of [Reynoso’s] concern for his health is dubious given that he rejected the opportunity to receive the COVID-19 vaccine.”^47^ In denying a motion for compassionate release, the court in *United States v. Cooper* reached a similar conclusion: “[I]f Defendant had any serious concerns or fears for his health, safety and well-being as a consequence of the coronavirus, he would have availed himself of the COVID-19 vaccine which was offered.”^48^ In *United States v. Broadfield*, the court determined that the petitioner’s remaining COVID-19 risk due to vaccine refusal was “self-incurred.”^49^ The court in *United States v. Pemberton* stated that it “[would] not reward Mr. Pemberton for turning down a vaccine that may save his life, reduce the potentially severe impact of COVID-19 infection on himself, and limit the risk he will spread the virus to others.”^50^ Finally, the court in *United States v. Greenlaw* described what it perceived to be the consequences of granting compassionate release for unvaccinated people: “To reward Mr. Greenlaw for his vaccination refusal would create a perverse incentive for defendants like Mr. Greenlaw to refuse COVID-19 vaccines and put their lives and the lives of others in jeopardy in an effort to bolster their compassionate release motions.”^51^

It is important to note that several courts took more neutral positions on COVID-19 vaccinations and compassionate release. Courts in *United States v. Moe*,^52^ *United States v. Sawyer*,^53^ *United States v. Russo*,^54^ and *United States v. Hartsell^55^* acknowledged that certain people might continue to face significant vulnerability to COVID-19 upon receiving the vaccine. In *United States v. Sawyer*, for example, the court acknowledged CDC guidance outlining continued COVID-19 risks for certain vaccinated people, commenting that “[o]ther courts have granted motions for compassionate release despite vaccinations because of concerns with effectiveness in individuals with obesity or because of other severe health conditions.” Based in part on these CDC guidelines, the court found that Sawyer’s case demonstrated extraordinary and compelling circumstances.^56^

### Reinfection

Courts additionally diverged on whether previous COVID-19 infection weighs against the “extraordinary and compelling” prong of the statute. Some courts have held that previous infections reduce the risk or severity of COVID-19-related concerns for compassionate release purposes. In an October 2021 opinion, *United States v. Rodrigues*, the court recognized Rodrigues’ increased risk should he be infected with COVID-19 again, but found that, because of his previous infection, “he has not established that he is likely to do so … [since] he already had COVID-19 in May 2020, reducing the likelihood of re-infection.”^57^ In *United States v. Baker*, the court acknowledged the argument that prior infection may not necessarily provide blanket protection from future illness. Ultimately, however, the court denied the petition, stating that the burden of establishing extraordinary and compelling circumstances falls to the petitioner, who did not “present[] any medical evidence showing that successful recovery from the COVID-19 virus fails to provide substantial protection from reinfection.”^58^ This case poses the question: how could petitioners reasonably prove their risk of severe reinfection?

Taking a different approach, courts in *United States v. Halliburton* and *United States v. Malauulu* acknowledged that previous infections do not eliminate a person’s risk for future infections. In *Halliburton*, the court cited the World Health Organization (WHO)’s warning that infection does not yield proven natural immunity against the virus, stating that “in addition to the very real risk of relapse or reinfection, Defendant also may suffer side effects from COVID-19.”^59^ In *Malauulu*, the court wrote that due to high facility infection rates, “lack of scientific certainty regarding whether reinfection is possible after a purported recovery or negative test, or whether COVID-19 immunity lasts, coupled with Mr. Malauulu’s prior infection and his multiple health conditions, this court errs on the side of caution and finds defendant’s specific risk of reinfection appears to be heightened.”^60^

In contrast to *United States v. Halliburton* and *United States v. Malauulu*, cases that minimize COVID-19 risk based on previous infections make unsubstantiated medical claims that invalidate COVID-19 concerns. Scientific understanding of the virus and immunity from previous infection evolved throughout the pandemic, but judicial opinions varied in their acknowledgement of this evolution. The court in *Halliburton* cites WHO’s warning as early as June 2020, indicating that judges were aware of concerns about pre-existing immunity even before reinfections were more prevalent due to the delta or omicron variants.^61^

However, in the context of the evolving pandemic and scientific understanding of COVID-19, decisions including *United States v. Rodrigues* and *United States v. Baker* have burdened petitioners with demonstrating that they are likely to face reinfection and severe COVID-19 symptoms.^62^ This theme calls for evidence that petitioners would face severe reinfection, which petitioners simply could not obtain as scientific understanding evolved and the public health community lacked consensus on the question of reinfection. As the pandemic continued to evolve, each subsequent variant has presented with different infectiousness and severity. Numerous studies reported enhanced severity in reinfected study participants,^63^ while others present no difference in severity^64^ or few severe outcomes,^65^ demonstrating the need for additional scientific evidence. Neither Baker nor Rodrigues could possibly have proven that they would be reinfected or that such a reinfection would be severe, but similarly, the government would not be able to prove the opposite. As a result, petitioners are left with the burden of demonstrating the impossible to access compassionate release.

### How “Extraordinary” Does “Extraordinary and Compelling” Need to Be?

#### Administrative Requirements

The First Step Act amendments to compassionate release allow petitioners to address courts directly in the event of BOP inaction. However, petitioners must first exhaust administrative remedies — waiting 30 days after requesting release from their warden before presenting their case to a judge.^66^ Some courts have outlined circumstances in which to waive this requirement. The court in *United States v. Barnes* identified three circumstances: “(1) the relief sought would be futile upon exhaustion; (2) exhaustion via the agency review process would result in inadequate relief; or (3) pursuit of agency review would subject the petitioner to undue prejudice.”^67^ Given the urgent threat of COVID-19, many petitioners sought to bypass the exhaustion requirement. However, many of these requests were denied for failing to meet the exhaustion timeline (see, e.g., *United States v. Ward*,^68^ *United States v. Rensing*,^69^ *United States v. Reese* ^70^). In fact, according to BOP data, 17.9%, 8.3%, and 5.7% of compassionate release denials were attributed to failure to satisfy the exhaustion requirement in 2020, 2021, and 2022, respectively.^71^ In *United States v. Britton*, for example, the court found that “the exhaustion requirement of § 3582(c)(1)(A) is a mandatory claim-processing rule and that the text and legislative history of the statute foreclose the applicability of equitable exceptions.”^72^

Other courts, however, have waived the 30-day waiting period, such as in *United States v. Lacy* and *United States v. Barnes*, each of which provided justifications for exceptions to the § 3582(c) requirement. In *Lacy*, the court determined that “§ 3582(c)(1)(A) does not require the Court to wait to consider a compassionate release request if there is a credible claim of serious and imminent harm from this pandemic,” deeming COVID-19 “serious and imminent” enough to forgo the requirement.^73^ The court in *Barnes* came to a comparable conclusion, stating that “[g]iven the exigencies created by COVID-19, the Court finds that exhaustion would be both futile and potentially subject defendant to undue prejudice. The Court therefore concludes that 3582(c)’s exhaustion requirement is appropriately waived.”^74^

Due to administrative process and judicial capacity, courts could not logistically decide compassionate release petitions immediately in every circumstance, and thus extreme cases would take priority to bypass exhaustion. However, in *United States v. Britton*, the court characterized the exhaustion requirement as “a mandatory claim-processing rule that is not subject to equitable exceptions,” diminishing the possibility for accelerated release, while courts in *United States v. Lacy* and *United States v. Barnes* deem acceleration appropriate under the circumstances of these cases.^75^ Thus, the differential determination of the flexibility of the exhaustion requirement created a notable discrepancy in pandemic compassionate release policies.

As is evidenced by the breadth of COVID-19-related compassionate release petitions, people seeking release have substantial differences in personal circumstances and COVID-19 risks. For some petitioners, these circumstances, combined with the requirement to wait 30 days, could mean the difference between life and death. Given that COVID-19 is a communicable disease, time spent in communal incarcerated settings would inevitably increase a person’s risk of contracting the virus. Thus, decisions that draw lines between cases fit for exceptionality and cases that are not have significant health implications for incarcerated people. If courts interpret the flexibility of exhaustion differently, as demonstrated in *United States v. Lacy* and *United States v. Britton*, petitioners face differing exposure-based risks of contracting COVID-19, even if they have otherwise equivalent risk profiles.

#### “Extraordinary” Conditions

Finally, the *United States v. Thompson* and *United States v. Mouton* decisions present extremely literal interpretations of the “extraordinary and compelling” requirement for compassionate release. Both decisions call into question the validity of petitioners’ requests due to the commonality of their comorbidities. In *Thompson*, the court “acknowledge[s] that Thompson’s chronic illnesses place him at a higher risk of severe symptoms, should he contract COVID-19,” but concludes that “nearly half of the adult population in the United States suffers from hypertension … [a]nd roughly 12% of Americans suffer from high cholesterol. Thus, we cannot say that either of those conditions makes Thompson’s case ‘extraordinary.’ Unfortunately, both are commonplace.”^76^ In *Mouton*, the court dismissed Mouton’s request because “a diagnosis of one or even several ailments comorbid with COVID-19 in conjunction with the threat of COVID-19 is not ‘extraordinary’ because such circumstances are ‘common.’”^77^

By requiring that reasons for release are not “common,” courts have concluded that comorbidities faced by high proportions of people in incarcerated settings discredit their request for release because too many people face high risks for COVID-19. In these circumstances, the question is no longer whether a person’s health and environmental circumstances elevate risk for poor COVID-19 outcomes, but whether their circumstances are unique enough to warrant release. These interpretations suggest that a person with health conditions dire enough to predict severe COVID-19 outcomes would not meet the requirements for compassionate release if enough of the population has the same comorbidities. Such a conclusion further undermines federal compassionate release policies by failing to protect incarcerated people if their conditions are common.

## Discussion of Ethical Considerations

The ethical implications of these inconsistencies are critical. Consider, by way of example, the inconsistent application of vaccination status. These compassionate release case decisions and their statements on vaccination present several ethical concerns, including their potential to promote vaccine opposition, dismissal of the pandemic’s continued danger, and dismissal of the risk of severe outcomes for people with preexisting health conditions. First, regarding vaccine opposition, it is important to consider the implication of characterizing the vaccine as a determinant against extraordinary and compelling circumstances for release. To be sure, COVID-19 vaccines have proven to protect against severe COVID-19 outcomes and therefore serve as effective and safe public health tools to prevent or end a pandemic.^78^ Thus, it would be logical to consider vaccine efficacy when determining COVID-19 risk. However, broad statements characterizing access to vaccines for “compassionate release motions generally lack[ing] merit” may have promoted vaccine opposition.^79^

Second, the courts seem to portray the vaccine as a panacea in the fight against COVID-19. Although the COVID-19 vaccine is a core prevention strategy, vaccinated people sometimes get infected with the virus that causes COVID-19. Current evidence indicates that a combination of core prevention strategies is the most effective strategy to reduce the risk of transmission. This is particularly true for older adults and people with severe health conditions. As the petitions themselves demonstrate, not everyone has the same risk profile for the virus, meaning that many people still face significant COVID-19 risks even when vaccinated. Though decisions such as *United States v. Moe* indicate that some federal judges factor appreciation for the continued risk of COVID-19 into their decision-making, in other cases,^80^ such as *United States v. Reed*, well-founded concerns about the ongoing pandemic are discounted.^81^ In dismissing an incarcerated individual’s concerns of contracting COVID-19 based solely on their vaccination status, courts fail to afford adequate consideration for the personal circumstances presented by each person and thus neglect to render the individualized determination demanded by proper consideration of a motion brought under the First Step Act. Thus, differing opinions on the limitations of vaccines have contributed to differing access to federal compassionate release.

Jurisdictions that have found that vaccination status disqualifies individuals from compassionate release set the law and public health in opposition. Such rulings undercut the well-documented risk of severe COVID-19 health outcomes for individuals with underlying medical conditions.^82^ As a result, incarcerated individuals may have chosen to forgo vaccination, putting themselves and others at greater risk of COVID-19 transmission and severe illness, perceiving it to be their best pathway to release.

The jurisdictional divide on how to weigh, interpret, and apply vaccination status to compassionate release cases may have additionally led to health disparities by jurisdictional location. Jurisdictions that have determined that choosing to remain unvaccinated is a self-inflicted risk may have seen higher vaccination rates as petitioners learned that they must be vaccinated for the court to consider their release. Jurisdictions that ruled that vaccinated people are no longer at risk may have seen lower vaccination rates as petitioners forgo vaccination in favor of a better chance at release.

While the situation at hand is somewhat limited to an extreme example — an unprecedented pandemic — these rulings may have had broader implications for how courts inadvertently create perverse incentives in how they assess preventive or health promotive steps when considering health consequences in compassionate release cases. For someone with an elevated risk of stroke, would regular exercise and dietary modifications be considered actions that, if undertaken in some jurisdictions, would render someone ineligible for release because they are healthier than they might otherwise have been, and in others be viewed as indications of how seriously the person perceives the risk of heart disease and stroke? Such divergence raises the question as to whether a jurisdiction itself should be viewed as a social determinant of health.

## Conclusion

As demonstrated by publicly available compassionate release court decisions, the COVID-19 pandemic produced inconsistent interpretations of compassionate release requests related to COVID-19 risk following the First Step Act. To be sure, weighing a person’s extraordinary and compelling reasons for release against their legal indiscretion, length of sentence, and other factors is highly individualistic due to differing circumstances, as is weighing a person’s health conditions against COVID-19 transmission risk and outcome. However, individual court decisions reveal concerning conclusions related to COVID-19 risks. Broad generalizations that the availability of COVID-19 vaccines invalidates COVID-19 compassionate release requests, previous COVID-19 infections invalidate concern of reinfection, and COVID-19 risk profiles must be uncommon are ethical misconceptions that have significant implications for the health and safety of federally incarcerated individuals. The 2023 policy amendments endorsed pandemic risk as a suitable reason for compassionate release.^83^ However, our examination of pandemic case law identified wide variation in courts’ application of public health considerations that resulted in differing access to compassionate release based on federal jurisdiction. Future application of the policy can consider the public health implications of such decisions.

## References

[1] M. Doering, “One step forward: compassionate release under the first step act,” Wisconsin Law Review 2020, no. 6 (2020): 1287–1320, https://wlr.law.wisc.edu/wp-content/uploads/sites/1263/2021/10/16-Doering-Final.pdf.

[2] J.E. James et al., “COVID-19 and the reimaging of compassionate release,” International Journal of Prisoner Health 19, no. 1 (2023): 20–34, 10.1108/IJPH-08-2021-0072. 35730723 PMC10134411

[3] *United States v. Pena*, 459 F. Supp. 3d 544 (S.D.N.Y. 2020).

[4] *United States v. Thompson*, 984 F. 3d 431 (5th Cir. 2021).

[5] See Doering, *supra* note 1; The Sentencing Reform Act simultaneously created the Sentencing Commission, a body of seven people nominated by the President and approved by Congress (United States Sentencing Commission). The Commission is tasked with “develop[ing] sentencing policies for the federal courts” and “serv[ing] as an information resource for Congress, the executive, the courts and the public on matters relating to federal crime and sentencing” (United States Sentencing Commission). The Commission has the power to determine criteria for “extraordinary and compelling” grounds for compassionate release, first set out in 2006. This “Guidelines Manual” for compassionate release came to include medical conditions, age, and family circumstances (18 U.S.C. § 3582(c)(1)(A)) (Milton 2021); “About,” United States Sentencing Commission, https://www.ussc.gov/about-page (last visited August 7, 2024); K. Milton, “Redeeming the Lost Generation: The Scope of the First Step Act and Compassionate Release in Revisiting Pre-Booker Sentencing,” Seton Hall Law Review 52, no. 1 (2021): 347, https://scholarship.shu.edu/shlr/vol52/iss1/8.

[6] *The Federal Bureau of Prisons’ Compassionate Release Program* (US Department of Justice, April 2013), https://oig.justice.gov/reports/2013/e1306.pdf.

[7] C.M. Berryessa, “Compassionate Release as a ‘Right’ in the Age of COVID-19,” American Journal of Bioethics 20, no. 7 (2020): 185–187, 10.2139/ssrn.3794242. 32716795

[8] J. Jefferson-Bullock, “Are you (still) my great and worthy opponent: compassionate release of terminally ill offenders,” UMKC Law Review 83, no. 3 (2015): 521–564, https://ssrn.com/abstract=2662664.

[9] *Id.*

[10] *2006 Federal Sentencing Guidelines Manual* §3E1.1 (United States Sentencing Commission, November, 2006).

[11] Id.; *United States Sentencing Commission Guidelines Manual 2021* §3E1.1 (United States Sentencing Commission, November, 2021).

[12] *United States Sentencing Commission Guidelines Manual 2021* §3E1.1 (United States Sentencing Commission, November, 2021).

[13] United States Congress, *First Step Act of 2018*, https://www.congress.gov/115/bills/s756/BILLS-115s756enr.pdf.

[14] See *United States Sentencing Commission Guidelines Manual 2021*, *supra* note 12; See *F*i*rst Step Act of 2018*, *supra* note 13.

[15] M.P. Greenblatt, “In Search of Judicial Compassion: The Cantu-Lynn Divide over Compassionate Release for Federal Prisoners,” *Columbia Human Rights Law Review* 52, no. 1 (2020), https://hrlr.law.columbia.edu/hrlr/in-search-of-judicial-compassion-the-cantu-lynn-divide-over-compassionate-release-for-federal-prisoners/.

[16] *United States Sentencing Commission Guidelines Manual 2023* §3E1.1 (United States Sentencing Commission, November, 2023).

[17] *Id.*

[18] 18. S.G. Prost and B. Williams, “Strategies to Optimize the Use of Compassionate Release From US Prisons,” American Journal of Public Health 110, no. S1 (2020): S25–S26, 10.2105/AJPH.2019.305434. 31967883 PMC6987939

[19] C.M. Berryessa, “Public Support for Using ‘Second Chance’ Mechanisms to Reconsider Long-Term Prison Sentences for Drug Crimes,” Federal Sentencing Reporter 34, no. 1 (2021): 71–79, 10.1525/fsr.2021.34.1.71; K. Nowotny et al., “COVID-19 exposes need for progressive criminal justice reform,” *American Journal of Public Health* 110, no. 7 (2020): 967–968, https://doi.org/10.2105/AJPH.2020.305707; J. Jefferson-Bullock, “Consensus, Compassion, and Compromise? The First Step Act and Aging Out of Crime,” *Federal Sentencing Reporter* 32, no. 2 (2021): 70–75, https://doi.org/10.1525/fsr.2019.32.2.70; J. Jefferson-Bullock, “A Little Child Shall Lead Them: Juvenile Justice, Aging Out, and the First Step Act,” *Tennessee Law Review* 87, no. 3 (2020): 569–628, https://ir.law.utk.edu/tennesseelawreview/vol87/iss3/3; R. O’Leary, “Compassionate Release and Decarceration in the States,” *Iowa Law Review* 107, no. 2 (2022): 621–676, https://ilr.law.uiowa.edu/print/volume-107-issue-2/compassionate-release-and-decarceration-in-the-states.

[20] See Doering, *supra* note 1; J.F. Ferraro, “Compelling compassion: Navigating Federal Compassionate Release after the First Step Act,” *Boston College Law Review* 62, no. 7 (2021): 2463–2514, https://bclawreview.bc.edu/articles/119; See Greenblatt, *supra* note 15; Sentencing — Statutory Interpretation — “United States v. Bryant: Eleventh Circuit Creates Split by Holding that the First Step Act Does Not Grant Courts the Authority to Determine What Circumstances Justify Compassionate Release,” *Harvard Law Review* 135, no. 4 (2022), https://harvardlawreview.org/print/vol-135/united-states-v-bryant/.

[21] E. Zunkel and J.M. Lessnick, “Putting the ‘Compassion’ in Compassionate Release: The Need for a Policy Statement Codifying Judicial Discretion,” Federal Sentencing Reporter 35, no 3, (2023): 164–174, 10.1525/fsr.2023.35.3.164; M. Price, “The United States Sentencing Commission, Compassionate Release, and Judicial Discretion: The 2022–2023 Amendment Cycle,” *Federal Sentencing Reporter* 35, no. 3 (2023):175–180, https://doi.org/10.1525/fsr.2023.35.3.175; C.J Merken and B.J. Harris, “Damn the Torpedos! An Unprincipled, Incorrect, and Lonely Approach to Compassionate Release,” *Cardozo Law Review* 44, no. 2 (2022): 477, https://cardozolawreview.com/damn-the-torpedoes-an-unprincipled-incorrect-and-lonely-approach-to-compassionate-release/.

[22] See Prost and Williams, *supra* note 18; See O’Leary, *supra* note 19.

[23] See Berryessa, *supra* note 19; D. Roper, “Pandemic Compassionate Release and the Case for Improving Judicial Discretion over Early Release Decisions,” Federal Sentencing Reporter 33, no. 1–2 (2020): 27–35, 10.1525/fsr.2020.33.1-2.27; Y. Bustillo, “Compassionate Release During Crises: Expanding Federal Court Powers,” *Yale Law & Policy Review* 40, no. 1 (2021): 223–275, https://yalelawandpolicy.org/compassionate-release-during-crises-expanding-federal-court-powers.

[24] S. Schotland, “Let them go! Compassionate release for disabled prisoners with chronic health conditions during the COVID-19 public health emergency,” Disability Studies Quarterly 41, no. 3 (2021), https://www.dsq-sds.org/index.php/dsq/article/view/8305/6183.

[25] *Underlying Conditions and the Higher Risk for Severe COVID-19* (Centers for Disease Control and Prevention, July 30, 2024) https://www.cdc.gov/covid/hcp/clinical-care/underlying-conditions.html. 34009770

[26] *Community, Work, and School* (Centers for Disease Control and Prevention, February 11, 2020), https://www.cdc.gov/coronavirus/2019-ncov/community/homeless-correctionalsettings.html.

[27] See *Underlying Conditions and the Higher Risk for Severe COVID-19*, *supra* note 25.

[28] K. Blakinger and J. Neff, “‘They let people die’: US prisons bureau denied tens of thousands compassionate release during Covid,” *Guardian* (US), (June 11, 2021), https://www.theguardian.com/us-news/2021/jun/11/us-prisons-bureau-compassionate-release-covid.

[29] See Greenblatt, *supra* note 15.

[30] See Roper, *supra* note 23.

[31] *MEMORANDUM FOR DIRECTOR OF BUREAU PRISONS* (Office of the Attorney General, March 26, 2020), https://www.justice.gov/file/1105491/dl.

[32] *United States v. Koons*, 2020 WL 1940570 (W.D. La. Apr. 21, 2020).

[33] *Compassionate Release: The Impact of the First Step Act and COVID-19 Pandemic* (United States Sentencing Commission, March 2022),https://www.ussc.gov/sites/default/files/pdf/research-andpublications/research-publications/2022/20220310_compassionate-release.pdf.

[34] *Benefits of Getting a COVID-19 Vaccine* (Centers for Disease Control and Prevention, September 22, 2023), https://www.cdc.gov/coronavirus/2019-ncov/vaccines/vaccine-benefits.html.

[35] *United States v. Lemons*, 15 F.4th 747 (6th Cir. 2021).

[36] Stanley-Becker, “Early Vaccination in Prisons, a Public Health Priority, Proves Politically Charged,” *Washington Post*, January 6, 2021, https://www.washingtonpost.com/health/2021/01/02/covid-vaccine-prisons/.

[37] L.M. Hagan et al., “COVID19 vaccination in the Federal Bureau of Prisons, December 2020–April 2021,” Vaccine 39, no. 40 (2021): 5883–5890, 10.1016/j.vaccine.2021.08.045. 34465473 PMC8363472

[38] *Id.*

[39] *United States v. Reed*, No. 18-CR-0078, 2021 WL 2681498 (E.D. Pa. June 30, 2021).

[40] *United States v. Rodrigues*, No. 16-CR-00529-DKW-KJM, 2021 WL 4927994 (D. Haw. Oct. 21, 2021).

[41] *United States v. Ueki*, No. 08-CR-00715-DKW, 2021 WL 2767062 (D. Haw. June 28, 2021).

[42] *United States v. Russo*, No. CR 20-47, 2021 WL 5792696 (E.D. Pa. Dec. 7, 2021).

[43] *United States v. Burrough*, No. 3:04-CR-00191-FDW, 2022 WL 2318512 (W.D.N.C. June 28, 2022).

[44] *United States v. Sanchez*, No. 19-CR-03775-BAS-1, 2021 WL 3371073 (S.D. Cal. Aug. 3, 2021).

[45] *United States v. Meza-Acosta*, No. 20-CR-01318-BAS-1, 2021 WL 3171977 (S.D. Cal. July 27, 2021).

[46] *United States v. Singh*, 525 F. Supp. 3d 543 (M.D. Pa. 2021).

[47] *United States v. Reynoso*, 525 F. Supp. 3d 253 (D. Mass. 2021).

[48] *United States v. Cooper*, No. CR 01-512-05, 2021 WL 1629258 (E.D. Pa. Apr. 27, 2021).

[49] *United States v. Broadfield*, 5 F.4th 801, 803 (7th Cir. 2021).

[50] *United States v. Pemberton*, No. 1:18-CR-00099-JAW, 2021 WL 2953670 (D. Me. July 14, 2021).

[51] *United States v. Greenlaw*, No. 1:18-CR-00098-JAW-06, 2021 WL 1277958 (D. Me. Apr. 6, 2021).

[52] *United States v. Moe*, 571 F. Supp. 3d 267 (D.N.J. 2021).

[53] *United States v. Sawyer*, No. 5:15-CR-160-BO-1, 2021 WL 3051985 (E.D.N.C. June 15, 2021).

[54] See *United States v. Russo*, *supra* note 42.

[55] *United States v. Hartsell*, No. 4:17-CR-195-DPM, 2022 WL 1127100 (E.D. Ark. Apr. 13, 2022).

[56] See *United States v. Sawyer*, *supra* note 53.

[57] See *United States v. Rodrigues*, *supra* note 40.

[58] *United States v. Baker*, No. 12-10076-01-JTM, 2021 WL 699935 (D. Kan. Feb. 23, 2021).

[59] *United States v. Halliburton*, No. 17-CR-20028, 2020 WL 3100089 (C.D. Ill. June 11, 2020).

[60] 60. *United States v. Malauulu*, No. 2:15-CR-00124-KJM, 2020 WL 7695588 (E.D. Cal. Dec. 28, 2020).

[61] See *United States v. Halliburton*, *supra* note 59.

[62] See *United States v. Rodrigues*, *supra* note 40.; See *United States v. Baker*, *supra* note 58.

[63] L.A. Dos Santos et al., “Recurrent COVID-19 including evidence of reinfection and enhanced severity in thirty Brazilian healthcare workers,” Journal of Infection 82, no. 3 (2021): 399–406, 10.1016/j.jinf.2021.01.020; N. Zucman et al., “Severe Reinfection with South African Severe Acute Respiratory Syndrome Coronavirus 2 (SARS-CoV-2) Variant 501Y.V2,” *Clinical Infectious Diseases* 73, no. 10, (2021): 1945–1946, https://doi.org/10.1093/cid/ciab129. 33589297 PMC7880834

[64] N.N. Nguyen et al., “SARS-CoV-2 reinfection and COVID-19 severity,” Emerging Microbes & Infections 11, no. 1 (2022): 894–901, 10.1080/22221751.2022.2052358. 35264078 PMC8942490

[65] S. Medić et al., “Risk and Severity of SARS-COV-2 Reinfections during 2020-2022 in Vojvodina, Serbia: A Population-Level Observational Study,” The Lancet Regional Health Europe 20, no. 100453, (2022), 10.1016/j.lanepe.2022.100453. PMC9246704 35791336

[66] See *First Step Act of 2018*, *supra* note 13.

[67] *United States v. Barnes*, No. 5:18-CR-347-BO-1, 2021 WL 190912 (E.D.N.C. Jan. 19, 2021). For those well versed in administrative law, these criteria may look familiar.

[68] *United States v. Ward*, No. 1:17CR68-LG-JCG-5, 2020 WL 4032245 (S.D. Miss. July 16, 2020).

[69] *United States v. Rensing*, No. 16 CR 442 (VM), 2020 WL 4003643 (S.D.N.Y. July 15, 2020).

[70] *United States v. Reese*, No. 12 CR 629 (VM), 2020 WL 2554381 (S.D.N.Y. May 20, 2020).

[71] *U.S. Sentencing Commission Compassionate Release Data Report* (United States Sentencing Commission, December, 2022), https://www.ussc.gov/sites/default/files/pdf/research-and-publications/federal-sentencing-statistics/compassionate-release/20221219-Compassionate-Release.pdf.

[72] *United States v. Britton*, 473 F. Supp. 3d 14 (D.N.H. 2020).

[73] *United States v. Lacy*, No. 15-CR-30038, 2020 WL 2093363 (C.D. Ill. May 1, 2020).

[74] See *United States v. Barnes*, *supra* note 67.

[75] See *United States v. Britton*, *supra* note 72; See *United States v. Lacy*, *supra* note 73; See *United States v. Barnes*, *supra* note 67.

[76] See *United States v. Thompson*, *supra* note 4.

[77] *United States v. Mouton*, No. 08-CR-363-07-07, 2022 WL 463314 (W.D. La. Feb. 15, 2022).

[78] See *Benefits of Getting a COVID-19 Vaccine*, *supra* note 34.

[79] See *United States v. Reed*, *supra* note 39.

[80] See *United States v. Moe*, *supra* note 52.

[81] See *United States v. Reed*, *supra* note 39.

[82] See *Underlying Conditions and the Higher Risk for Severe COVID-19*, *supra* note 25.

[83] See *United States Sentencing Commission Guidelines Manual 2023*, *supra* note 16.

